# 10.A. Workshop: Integrated primary care: reform experiences from Estonia, Poland, and Slovenia

**DOI:** 10.1093/eurpub/ckac129.623

**Published:** 2022-10-25

**Authors:** Organised by: European Observatory on Health Systems and Policies, Chair persons: Ewout Van Ginneken (Germany), Katherine Polin (Germany)

## Abstract

The rising burden of multiple chronic diseases requires countries to change the reactive approach of their health systems, originally designed to cure acute illness, to a proactive approach that scales up preventive interventions and integrates cure (health care) and care (long term care and social care). This requires a central coordination role for primary health care (PHC). PHC is the foundation for delivering health services to the population. It mainly encompasses the first level of professional health care and includes PHC practitioners and other health workers. Together, PHC providers can satisfy most curative and preventative health needs, ideally providing integrated services. In this way, PHC has the potential to agilely respond to population health needs; help individuals navigate their way to good health and stay healthy; prevent disease by identifying risk factors; and manage chronic disease outside of hospital settings. Yet PHC in many countries is not designed to fully realize these promises and ongoing reforms aim to strengthen it. Meanwhile, this potential was on display in several countries during the COVID-19 pandemic, when primary care doctors played a key role in making an early diagnosis; helping vulnerable people cope with their anxiety about the virus; reducing the demand for hospital services; and delivering vaccinations-all while reaching out to vulnerable patients and maintaining access to essential (non-COVID) services for the wider population using new in-person protocols or new digital solutions. This workshop will update the audience on recent PHC reforms across the EU and provide insights from three key examples-Estonia, Poland, and Slovenia. An introductory presentation sets the stage by putting PHC reforms in a wider context of reforms introduced across the 31 countries of the Health Systems and Policy Monitor network of the European Observatory on Health Systems and Policies between 2018 and 2021. A second presentation describes how Estonia has introduced a series of PHC reforms over time and used financial incentives to encourage multidisciplinary care. A third presentation looks to Poland, which from mid-2018 through 2021 piloted a multi-professional model of PHC organization in around 40 PHC practices, with an emphasis on care coordination and disease prevention. A final presentation highlights Slovenia, where a multidisciplinary, community-based, prevention-oriented service delivery model is being expanded to play a larger role in the prevention and management of chronic diseases as well as the country's COVID-19 response. The workshop concludes with an audience discussion on lessons learned from the three case studies and potential implications for other countries.

**Key messages:**

• Strengthening primary health care is a key priority of many countries as they seek to improve integration of care.

• Implementation of primary care reforms in shaped by the legacy of care delivery and requires a strong workforce, ongoing financial support, sustained policy focus, and collaborative governance.

